# Role of Bioimpedance Phase Angle and Hand Grip Strength in Predicting 12-Month Mortality in Patients Admitted with Haematologic Cancer

**DOI:** 10.3390/cancers17050886

**Published:** 2025-03-05

**Authors:** Lara Dalla Rovere, Rocio Fernández-Jiménez, Alessandro Guerrini, María García-Olivares, Cristina Herola-Cobos, Carmen Hardy-Añón, Rahinatu Awol-Tanko, Agustín Hernandez-Sanchez, José Manuel García-Almeida

**Affiliations:** 1 Department of Endocrinology and Nutrition, Quironsalud Malaga Hospital, 29004 Malaga, Spain; lara92net@gmail.com (L.D.R.); jgarciaalmeida@gmail.com (J.M.G.-A.); 2 Department of Medicine and Dermatology, Faculty of Medicine, University of Malaga, 29010 Malaga, Spain; 3 Instituto de Investigación Biomédica de Málaga y Plataforma en Nanomedicina-IBIMA Plataforma BIONAND, 29590 Malaga, Spain; 4 Department of Endocrinology and Nutrition, Virgen de la Victoria University Hospital, 29010 Malaga, Spain; 5 IRCCS Fondazione Don Carlo Gnocchi ONLUS, 50143 Florence, Italy; 6 Department of Science and Technology for Humans and the Environment, Università Campus Bio-Medico di Roma, 00128 Rome, Italy; 7 Department of Endocrinology and Nutrition, Regional de Málaga University Hospital, Málaga Biomedical Research Institute and BIONAND Platform (IBIMA), 29010 Malaga, Spain; 8 Department of Haematology, Quironsalud Malaga Hospital, 29004 Malaga, Spain; 9 Department of Endocrinology and Nutrition, Hospital Universitario Virgen de la Victoria, CIBEROBN, Carlos III Health Institute (ISCIII), University of Málaga, 29016 Malaga, Spain

**Keywords:** bioimpedance, phase angle, hand grip strength, haematologic cancer, nutritional assessment, mortality prediction, body composition

## Abstract

**Background/Objectives**: Haematologic cancers, such acute leukaemia, lymphoma, and multiple myeloma, are associated with high morbidity and mortality rates, often exacerbated by malnutrition and functional decline. This study aims to evaluate the prognostic value of bioimpedance phase angle (PhA) and hand grip strength (HGS) as nutritional and clinical markers for predicting 12-month mortality in hospitalized patients with haematologic cancers. **Methods**: A retrospective observational study was conducted on 121 patients admitted to Hospital Quironsalud Málaga between January 2019 and June 2021. PhA was measured using bioelectrical impedance analysis (BIA) and HGS was assessed using a dynamometer. Nutritional status was evaluated through Subjective Global Assessment (SGA) and the Global Leadership Initiative on Malnutrition (GLIM) criteria. The primary outcome was 12-month mortality, analysed using ROC curves, Kaplan–Meier survival estimates, and multivariate logistic regression models. **Results**: Lower PhA (<3.8° for females, <5.4° for males) and reduced HGS (<17 kg for females, <28 kg for males) were significantly associated with higher 12-month mortality (*p* < 0.001). The optimal PhA cut-off showed high sensitivity (85.5%) and specificity (62.2%). Multivariate analysis confirmed PhA as an independent predictor of mortality (OR = 0.417, *p* = 0.023). Patients with lower PhA and HGS values exhibited reduced survival rates, emphasizing the importance of these markers in clinical practice. **Conclusions**: PhA and HGS are reliable, non-invasive tools for assessing prognosis in haematologic cancer patients. Incorporating these markers into routine care could improve risk stratification, guide nutritional interventions, and enhance patient outcomes.

## Simple Summary

This study evaluates the use of bioimpedance phase angle (PhA) and handgrip strength (HGS) as predictors of survival in patients with haematologic cancers. These non-invasive and accessible tools provide essential information about patients’ nutritional status and overall health. Our findings reveal that lower PhA and reduced HGS are significantly associated with higher 12-month mortality rates. By integrating these markers into routine clinical practice, healthcare providers can improve risk stratification and develop personalised nutritional and therapeutic strategies to enhance patient outcomes.

## 1. Introduction

Haematological pathologies, including leukaemia, lymphoma, and multiple myeloma, represent a significant global health challenge, with rising incidence and mortality rates [1]. This emphasises the need for improved prognostic tools and more precise nutritional assessment methods, designed specifically for these patients. Malnutrition is a common complication in individuals with haematologic cancer, driven both by the catabolic effects of the malignancy and by the exacerbation caused by aggressive treatments. The result is often notable weight and muscle loss and reduced functional capacity [2,3]. An accurate nutritional assessment is crucial in this context, as it offers valuable insights into overall clinical conditions and allows for more precise targeted intervention strategies to be chosen [4].

Although conventional nutritional markers like body mass index, albumin levels, and weight loss have been evaluated in patients with haematologic cancer, they often fail to capture the full complexity of nutritional changes [5]. In response, the Morphofunctional^®^ assessment has emerged, focusing on both body composition (“Morpho”) and functionality (“Functional”), based on ESPEN’s definition of malnutrition, which emphasises the interplay between under- or overnutrition and inflammation [6]. Among the techniques used in Morphofunctional^®^ assessment, we have the vectorial bioelectrical impedance analysis (BIVA) and hand grip strength as predictive markers representing a promising approach [7,8].

The phase angle (PhA) is a value directly derived from bioelectrical impedance analysis (BIA). It represents the ratio between reactance, which indicates the integrity of cell membranes, and resistance, which reflects the opposition of body fluids to electrical current flow. PhA is used to assess cellular health and body composition, with higher values suggesting better cellular integrity and lower values indicating possible cell damage or malnutrition [9]. The PhA seems to be a good indicator for mortality [9]. It is strongly associated with health and nutritional status, as it is indicative of both the integrity of cellular membranes and the cellular water distribution [10]. BIA is able to indirectly estimate body composition and serves as a valuable, non-invasive tool for nutritional assessment in hospitalised patients [11]. Its association with inflammatory and oxidative stress emphasises its effectiveness in evaluating the nutritional status and overall health of individuals with haematologic cancer [12,13].

Similarly, hand grip strength (HGS), an indicator of muscle strength and overall functional capacity, has emerged as a predictive marker for clinical outcomes in cancer patients [14]. Unlike other nutritional prognostic tools, hand grip strength has demonstrated a good predictor of adverse clinical outcomes and mortality in patients with blood cancer [14,15]. The support from the European Society for Clinical Nutrition and Metabolism underscores its relevance in identifying malnutrition among this patient population [4].

The use of phase angle and hand grip strength as Morphofunctional^®^ techniques and predictive markers shows considerable potential in addressing the nutritional challenges in haematologic cancer patients, particularly due to muscle mass loss [16]. These markers provide important insights into overall health status and loss in muscle mass, and this can inform personalized nutritional interventions.

Moreover, the non-invasive and easily accessible nature of BIA and HGS makes them viable options for routine clinical utilization [5,8]. Their capacity to provide valuable information on nutritional status and overall health makes them reliable choices for assessment in haematologic cancer patients [17].

The aim of this study is to evaluate the predictive value of bioimpedance phase angle and hand grip strength as nutritional markers in haematologic cancer patients.

## 2. Materials and Methods

### 2.1. Setting Study

In this retrospective observational study conducted at a single centre, patients diagnosed with haematological cancers and admitted to Hospital Quironsalud Malaga for various reasons were evaluated within 24 h. Bioelectrical impedance analysis (BIA), anthropometric measurements, and functional tests like hand grip strength (HGS) were conducted from January 2019 to June 2021. The study excluded paediatric patients, pregnant women, and short-stay patients. All patients underwent SGA (Subjective Global Assessment) evaluation, as the gold standard for diagnosing malnutrition, alongside the use of the new GLIM (Global Leadership Initiative on Malnutrition) criteria for early malnutrition diagnosis [3,18].

This study was approved by the Ethics Committee of Hospital Regional Malaga (2758-N-21). All eligible patients who agreed to participate through informed consent were included within 48 h of admission. No exclusion criteria were applied; there were no patient refusals and no inability to undergo BIA measurements due to various reasons such as ethnicity-related issues or physical limitations.

A flow chart diagram shows how patients were selected for our study (Supplementary Figure S1).

### 2.2. Anthropometrical, Phase Angle, and Other Parameters of Bioelectrical Impedance Analysis

Bioelectrical impedance was measured within the first 24–48 h after hospital admission with NUTRILAB, a phase-sensitive touch screen impedance device (Nutrilab, Akern Srl, Florence, Italy), through working with an alternating sinusoidal electric current of 245 microampere at an operating frequency of 50 kHz (±1%). The device was calibrated every morning using the standard control circuit supplied by the manufacturer with a known impedance resistance (R) = 380 ohm; reactance (Xc) = 45 ohm [19]. For the BIA measurement, each participant was moved into a supine position with both limbs slightly spread apart from the body. Very-low-intrinsic-impedance (<30 ohm) disposable electrodes (BIATRODES Akern Srl; Florence, Italy) were placed on the right or left side at metacarpal and metatarsal sites of the same wrist and ankle. PhA was expressed in degrees as arctan (Xc/R) × (180°/π). A standardised PhA value was obtained by normalising the observed PhA against age- and sex-specific reference values, providing a measurement of cell membrane integrity and body cell mass [11]. Consistent protocols reinforced measurement precision, with measurements taken after a five-minute stabilization period to minimize the impact of fluid shifts on impedance values.

### 2.3. Functional Parameters

We assessed hand grip strength using a JAMAR hand dynamometer (Model BK-7498, Fred Sammons Inc., Brookfield, IL, USA). Grip strength was evaluated while seated with the elbow flexed at 90 degrees. Patients were instructed to perform three maximal isometric contractions with short breaks between measurements, and the median value was recorded.

### 2.4. Nutritional and Clinical Variables

Demographic characteristics, comorbidities, MUST (Malnutrition Universal Screening Tool) and SGA assessment, GLIM criteria, and clinical and anthropometric data along with other nutritional evaluations were gathered. A skilled nutritionist assessed the participants’ malnutrition risk using the MUST score based on current body mass index, documented weight loss (defined as the percentage of body weight lost over the six months prior to hospital admission), and the presence of acute illness or no nutritional intake in 5 days. This classified patients as 0 (no-risk), 1 (moderate-risk), or 2 (high-risk). Patients were also categorised using SGA into well-nourished, moderately malnourished, or severely malnourished groups. Additionally, the identification of malnutrition according to the criteria from the Global Leadership Initiative on Malnutrition (GLIM criteria) and was classified based on severity as stage 1 (moderate) or stage 2 (severe malnutrition).

### 2.5. Clinical Outcomes

The primary objective was to evaluate the predictive significance of PhA for the risk of mortality in hospitalised haematological cancer patients. The secondary goal was to assess the prognostic value of PhA and HGS for mortality, registering death within a year of their evaluation.

### 2.6. Sample Size Calculation

Our aim was to investigate whether PhA and SphA can independently predict 12-month mortality in HC patients. Sample size calculation was based on the results of Garlini et al. and previous studies using different predictor variables [9]. With an alpha error of 0.05, a power of 80%, and a loss rate of 10%, we determined that at least 108 patients were required to achieve an adequate statistical power. Consequently, our target recruitment number was set at 121 patients [9].

### 2.7. Statistical Analysis

Our data were analysed using the JAMOVI software (version 2.5.3, The Jamovi Project, Sydney, Australia), starting with descriptive statistics to characterise the cohort. The Shapiro–Wilk test checked the normality of quantitative distributions. Associations between survival and non-survival were examined using *t*-tests, Mann–Whitney U tests, and chi-squared tests, as appropriate. The prognostic utility of PhA, SphA, and HGS for 12-month mortality was assessed through ROC curve analysis, with the area under the curve (AUC) indicating each test’s discriminative power.

To quantify the cumulative probability of mortality and assess survival rates across various phase angle (PhA) mortality and survival cut-off points during a 12-month period, we employed the Kaplan–Meier product-limit estimator. Differences in survival distributions were statistically evaluated using the log-rank test. The day of admission was designated as the starting point for analysis, with death marked as the endpoint event; all cases were censored at the last known date of follow-up. A *p*-value < 0.05 was considered statistically significant in determining the presence of significant differences.

For the multivariate statistical analysis, a binomial logistic regression was conducted to identify predictors of 12-month mortality in hospitalised patients with HC. The predictors included in the model were selected based on their clinical relevance and preliminary analysis results. The dependent variable was 12-month mortality (dichotomous variable: 1 = deceased, 0 = survival).

Phase angle (PhA) and hand grip strength (HGS) were stratified by sex rather than age, following standard reference values in the literature, as bioelectrical impedance parameters and muscle strength differ primarily by sex. Age was included as a covariate in the multivariate analysis to account for its potential influence on these functional and nutritional markers in relation to mortality.

Binomial logistic regression was used to estimate odds ratios (OR) and their respective 95% confidence intervals (95% CI) for each predictor. Model fit was assessed using McFadden’s pseudo R^2^ and the Akaike Information Criterion (AIC). The overall significance of the model was evaluated with the likelihood ratio chi-square test. The discriminative ability of the model was assessed by calculating the area under the curve (AUC) of the ROC curve.

## 3. Results

### 3.1. General Characterization of the Population Study

In this study, we analysed data from 121 patients diagnosed with haematological cancers who were admitted to the hospital for various reasons. The cohort had an average age of 64 ± 15 years, with a slight majority being female (54.5%, n = 66).

The distribution of haematologic malignancies within the study population varied, and lymphoma (aggressive and indolent) was diagnosed in 62 patients (52.5%), making it the dominant cancer type. This was followed by acute leukaemia in 32 patients (27.1%), multiple myeloma in 20 patients (17%), and amyloidosis in 4 patients (3.4%). In terms of treatment, most patients were undergoing their first line of chemotherapy, accounting for 68.6% of the cohort. However, 38 patients (31.4%) had progressed to a second line of treatment, indicative of either relapse or a refractory response to the initial therapy. The significant proportion of patients on second-line treatments underscores the challenges in managing haematologic malignancies effectively, highlighting the importance of evaluating prognostic factors such as PhA and HGS in anticipating patient outcomes.

Analysis of the haematological cancer subtypes encountered in the patient cohort are shown in Supplementary Table S1. Diffuse Large B-Cell Lymphoma (DLBCL) was the most prevalent, representing 25.4% of the total cases. This was followed closely by Acute Myeloid Leukaemia (AML), which accounted for 22% of the cases. Multiple myeloma also represented a substantial portion of the cohort. This prevalence indicates a notable occurrence of this cancer type, which is known for its chronic nature and complex treatment needs. The data reveal less common types of haematological malignancies such as T/NK-cell T/NK Non-Hodgkin Lymphoma and Mantle Cell Lymphoma, which are also present, though in smaller numbers, suggesting a range of lymphoproliferative disorders that require specialized attention.

The comprehensive breakdown provided by a table allows for a deeper understanding of the haematological landscape within the study, offering insights into the distribution of cancer subtypes and their relative frequencies. This detailed characterization aids in assessing the specific needs and prognostic factors relevant to each subtype, crucial for personalized treatment approaches and outcome research.

A minority of the cohort, constituting 22.5%, comprises patients who have been recently diagnosed and are in the initial stages of their therapy. This early phase of treatment is critical as it sets the groundwork for disease management strategies and initial therapeutic responses. The most substantial portion of the study population, 45.8%, included individuals who achieved remission, having responded favourably to their initial or subsequent therapeutic interventions. The remission phase is crucial as it indicates successful disease control and underscores the effectiveness of the treatment modalities employed. Additionally, 31.7% of the participants were categorized within the relapse or refractory stage. Patients in this phase were experiencing either a resurgence of the disease or a lack of response to established treatment protocols. This stage often requires the implementation of more aggressive and complex treatment strategies.

Nutritional assessments revealed a high prevalence of malnutrition: 73.6% of patients were identified as malnourished under the Subjective Global Assessment (SGA) criteria (SGA B and C), and 58% met the criteria for moderate to severe malnutrition according to the Global Leadership Initiative on Malnutrition (GLIM 1 and 2).

The mean phase angle (PhA) across the entire sample was 4.5 ± 1.3°. Upon examining gender differences, significant disparities were noted in PhA values; males exhibited higher PhA measurements (5.0 ± 1.2°) compared to females (4.1 ± 1.1°, *p* < 0.001). The population’s hand grip strength (HGS) showed a significant difference, with males having a median of 34.22 ± 8.66 kg and females 20.42 ± 4.53 kg (*p* < 0.001).

In this population, significant differences were observed in phase angle (PhA) and hand grip strength (HGS) between survivors and non-survivors. Survivors had a higher median PhA (4.96° vs. 3.7°, *p* < 0.001) and greater HGS (29.4 kg vs. 17.7 kg, *p* = 0.022) compared to non-survivors.

In addition, the standardized phase angle (SPhA) is a variation of the traditional phase angle (PhA) that accounts for age and sex differences, allowing for more standardized comparisons across populations. Unlike raw PhA, which may be influenced by demographic factors, SPhA provides a normalized measure that enhances its applicability in diverse clinical settings. By adjusting for these variables, SPhA enables a more accurate assessment of cellular health and nutritional status, making it a valuable tool for evaluating prognosis in heterogeneous patient groups. The SPhA also showed a significant difference between survivors and non-survivors. Survivors had a higher median SPhA (−0.65) compared to non-survivors (−1.92), with a *p* < 0.001. In our cohort, the Skeletal Muscle Index (SMI) did not show statistically significant differences between survivors and non-survivors. Therefore, it was not considered a key predictor of 12-month mortality.

Demographic characteristics, anthropometric measurements, functional test results, and patients’ measurements by survival or not are shown in Table 1. The characteristics by gender and by the line of treatment are shown in Supplementary Table S2.

### 3.2. Correlation Analysis of Bioimpedance and Muscle Strength with Different Clinical Markers Indicative of Nutritional Status

Correlation analyses were employed to examine the associations between phase angle (PhA), hand grip strength (HGS), and different clinical markers indicative of nutritional status.

Table 2 shows the correlation analysis, which revealed a moderate positive relationship between hand grip strength (HGS) and phase angle (PhA), with a Spearman’s rho of 0.378, which was statistically significant (*p* < 0.01). This suggests that greater muscle strength, as indicated by HGS, is moderately related with better cellular integrity and overall cell function as represented by PhA. This relationship does not hold with SPhA.

### 3.3. PhA and Mortality

In examining the role of BIA parameters as potential prognostic indicators, a notable difference was observed between patients who survived and those who did not (Table 3). Notably, the data indicate that at the time of admission, patients who subsequently did not survive exhibited significantly lower values of both PhA and SPhA compared to those who survived. Specifically, non-survivors had a mean PhA of 3.7 ± 1.1°, markedly lower than the 5.0 ± 1.2 observed in survivors (*p* < 0.001). Similarly, the mean SPhA was significantly lower in non-survivors (−1.9 ± 1.3) compared to survivors (−0.6 ± 1.6, *p* < 0.001). These findings suggest that reduced PhA and SPhA measurements may be strong indicators of higher mortality risk, potentially serving as early markers for poor prognosis in patients with haematological malignancies.

**Figure 1:**
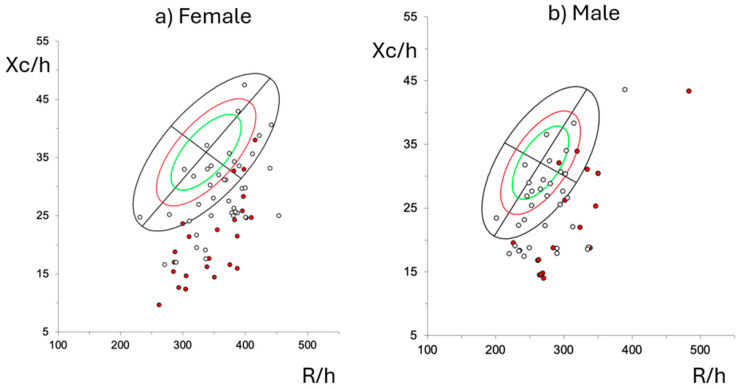
Clinical outcomes related to phase angle in haematological cancers patients, with red points indicating mortality cases. The ellipses represent the 50th, 75th, and 95th percentiles of normality, indicating reference ranges for bioimpedance measurements. R: resistance; H: height; Xc: reactance.

Figure 1 illustrates the bioelectrical impedance vector analysis (BIVA) distribution of patients with hematological malignancies, stratified by sex (panel a: female, panel b: male). The Xc/h (reactance normalized by height) and R/h (resistance normalized by height) are plotted to assess the differences in body composition and cellular integrity between groups. The red dots indicate patients who did not survive, emphasizing the relationship between bioimpedance parameters and mortality outcomes. Non-survivors tend to cluster toward the lower left region, which corresponds to lower reactance (Xc) and higher resistance (R)—both indicative of worse cellular integrity and reduced body cell mass.

**Figure 2:**
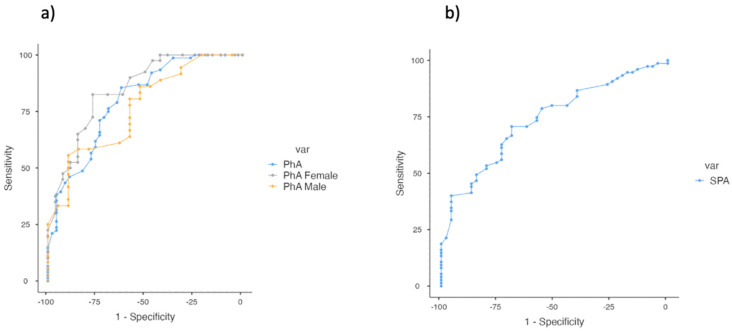
ROC curve analyses for PhA and SPhA variables to predict 12-month mortality. (**a**) Overall and gender ROC curve analysis for PhA. (**b**) ROC curve analysis for SPhA.

Using ROC curve analysis, we identified the optimal cut-off points for phase angle (PhA) and standardized phase angle (SPhA) to predict 12-month mortality (Figure 2).

ROC curve analysis showed that PhA had a significant discriminative ability to predict 12-month mortality (AUC = 798) among admitted patients.

The optimal cut-off point identified for PhA was 3.8° (85.5% sensitivity and 62.2% specificity). The Positive Predictive Value (PPV) and Negative Predictive Value (NPV) for this group were 79.3% and 71.8%, respectively, reflecting a robust predictive performance. For male patients, the cut-off for PhA was higher at 5.4° (55.6% sensitivity and 89.5% specificity) and the AUC was slightly lower at 0.762, suggesting good predictive accuracy, though not as strong as the overall group. In contrast, female patients showed superior predictive accuracy with an AUC of 0.845. The cut-off point remained at 3.8° (82.5% sensitivity and 76.9% specificity) (Table 4).

In addition, the overall SPhA cut-off for 12-month mortality was −1.5 (70.7% sensitivity and 68.9% specificity). The Area Under the Curve (AUC) for SPhA was 0.737, indicating a good ability to discriminate between patients who will and will not experience mortality within 12 months, though it was slightly less effective than the non-standardized phase angle (PhA).

**Figure 3:**
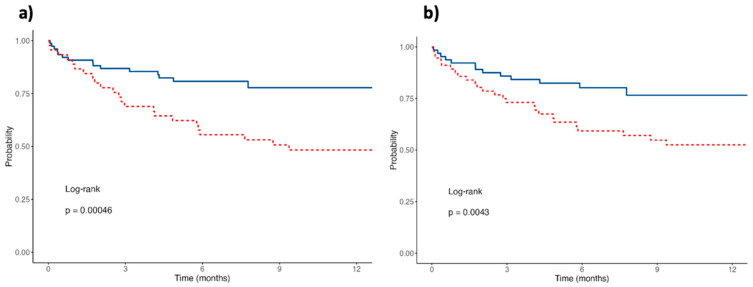
Kaplan–Meier survival curves of patients stratified by PhA and SPhA. (**a**) Survival curves for patients with PhA ≥ 3.8° (solid blue line) and PhA < 3.8° (dotted red line). (**b**) Survival curves for patients with SPhA ≥ −1.5 (solid blue line) and SPhA < −1.5 (dotted red line).

The Kaplan–Meier survival curves and corresponding log-rank tests for the PhA and SPhA cut-off are illustrated in Figure 3. The analysis indicates that patients with a PhA below the established cut-off of 3.8° have a 12-month survival rate of 48% (95% CI: 36–66). Similarly, those with an SPhA below the cut-off of −1.5 have a 12-month survival rate of 53% (95% CI: 40–68), which is lower compared to patients with higher SPhA values. These findings suggest that lower PhA (<3.8°) and SPhA (<−1.5) values are associated with poorer survival outcomes.

### 3.4. HGS and Mortality

Moreover, hand grip strength (HGS) was also analysed, revealing significant cut-off points to predict 12-month mortality at 28 kg for men (sensitivity 88.89%, specificity 40%) and 17 kg for women (sensitivity 95.24%, specificity 28.57%). The AUCs were 0.698 for men and 0.568 for women (Table 5).

**Figure 4:**
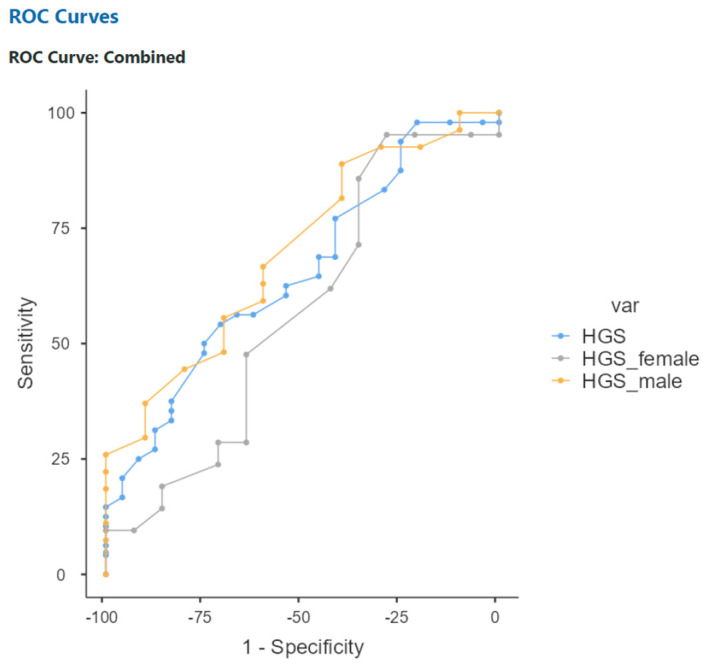
ROC curve analyses for HGS variables to predict 12-month mortality.

**Figure 5:**
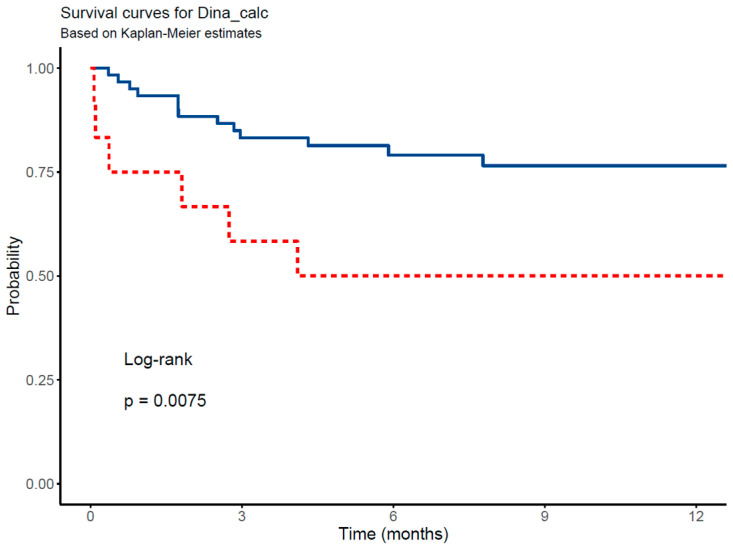
Kaplan–Meier survival curves of patients stratified by HGS. The solid blue line represents patients with HGS ≥ 28 kg for men and ≥17 kg for women, while the dotted red line represents patients with HGS < 28 kg for men and <17 kg for women.

These results, further supported by the Kaplan–Meier survival analysis shown in Figure 4 and Figure 5, demonstrate that patients with HGS values below the identified cut-offs have significantly lower 12-month survival rates. Specifically, the 12-month survival rate is 50% (95% CI: 28–88%) for women and 77% (95% CI: 66–88%) for men.

### 3.5. Multivariate Analyses for Predicting 12-Month Mortality

Furthermore, multivariate analyses were conducted to assess the prognostic value of these cut-offs in predicting 12-month mortality among hospitalized patients with HC, as detailed in Table 6 in the multivariate analysis to predict 12-month mortality in hospitalised patients with haematological cancers. Among the predictors, treatment line and phase angle (PhA) were statistically significant. Patients on a second line of treatment had a higher probability of mortality compared to those on a first line (OR = 14.245, *p* < 0.001). Additionally, a higher PhA was associated with lower mortality, with an OR of 0.417 (*p* = 0.023). Other factors, such as sex, age, and hand grip strength (HGS), did not show statistical significance in this model.

This model correctly predicted 85.1% of non-mortality cases and 62.5% of mortality cases. Regarding predictive metrics, the model achieved an accuracy of 0.775, a specificity of 0.851, and a sensitivity of 0.625, with an area under the curve (AUC) of 0.876. The model showed a McFadden pseudo R^2^ of 0.374 and a globally significant result (chi-square = 34.0, *p* < 0.001).

## 4. Discussion

This study demonstrates that both phase angle (PhA) and hand grip strength (HGS) are significant prognostic markers for 12-month mortality in hospitalised patients with haematologic cancer. In line with previous studies, we found that lower PhA values are associated with higher mortality, reinforcing the metric’s utility as a clinical indicator. Many studies have already documented the relationship between phase angle (PhA) and mortality in cancer patients. For instance, Genton et al. indicated that a low phase angle, especially in the lowest quartile, is associated with a higher risk of mortality, regardless of the BIA device used [20].

Our cohort showed consistency with the literature, showing that patients with a PhA below 3.8° had a significantly higher risk of mortality, and that a low PhA was repeatedly linked to poor clinical outcomes. Regarding cutoff points, various studies have suggested specific PhA values to be associated with a higher mortality risk. In particular, Norman et al. proposed that a PhA below 5.4° in men and 5.0° in women was associated with a significantly higher risk of mortality in cancer patients [10]. Our cutoff points were slightly lower than those suggested in other studies, which might have been due to differences in study populations, types of cancer, and disease stages. Another study conducted by Szeja and Grosicki in patients with lymphoproliferative neoplasms reported that a PhA below 4.5° was significantly predictive of adverse outcomes, including mortality and severe treatment complications [2]. This finding suggests that patients with a PhA below this threshold may require more intensive interventions and closer monitoring during treatment.

In our sample, a high prevalence of malnutrition was observed, particularly amongst women and those in advanced stages of treatment. These findings are consistent with those reported by Genton et al., who also identified that women and older patients were more likely to present severe malnutrition, potentially due to lower cellular mass and energy reserves [20]. Additionally, in agreement with the results of Szeja and Grosicki, our study confirms that patients in more advanced lines of treatment have a higher risk of malnutrition and poorer parameters such as PhA and HGS [2]. These data underscore the importance of conducting detailed nutritional and functional assessments, especially in vulnerable patients, to improve prognosis and the quality of life during cancer treatment. Furthermore, our findings highlight the significant impact of treatment line on patient outcomes, with second-line treatment emerging as the strongest predictor of mortality. This suggests that disease progression and prior treatment exposure play a critical role in survival. These results align with previous research, indicating that patients requiring second-line therapies have poorer prognoses due to reduced treatment efficacy and higher rates of complications.

In our cohort, the overall SPhA cut-off for 12-months mortality was −1.5, which is similar to other studies, where low sPhA has been related to mortality risk. In terms of cut off points, Paiva et al. suggested that cancer patients with SPA < −1.65 had a smaller survival rate than those with SPA  ≥  −1.65 [21].

As for hand grip strength (HGS), its utility as a predictor of mortality and complications in cancer patients has been established. Sealy et al. found that an HGS below 20 kg in women and 30 kg in men was associated with a higher likelihood of chemotherapy-related toxicity and early treatment discontinuation [16]. These cutoff points are similar to those used in our study, where HGS values also showed a significant correlation with mortality. The use of HGS as an indicator of functional capacity has already been documented in several research papers. An analysis by Arab et al. highlighted that HGS strongly correlates with muscle mass and overall functionality, and values below the established cutoffs are associated with increased mortality across various oncological populations [22].

This study reinforces the importance of HGS as a simple yet powerful tool for assessing risk in patients with haematologic cancer.

Collectively, these studies underscore the importance of using both PhA and HGS in the prognostic evaluation of patients with haematologic cancer. The cutoff points identified in these studies provide a valuable guideline for risk stratification and the planning of more personalised therapeutic interventions. The consistency of findings across different studies and populations further strengthens the robustness of these markers as essential tools in clinical practice [22]. A significant strength of our study is the use of multiple functional and nutritional assessment parameters, allowing for a comprehensive view of the patients’ status. Despite the strengths of this study, some limitations should be acknowledged. First, the retrospective nature of the analysis may have introduced selection bias and limited the availability of certain oncological variables, such as detailed staging systems and treatment-related toxicities. While patients were classified according to treatment line (first-line vs. second-line therapy) as an indirect indicator of disease severity and treatment intensity, we recognise that a more detailed classification of malignancies could provide additional prognostic insights. Furthermore, our study does not differentiate between specific chemotherapy regimens, stem cell transplantation, or treatment-related complications, which are known to influence survival outcomes. Future prospective studies should further explore the impact of different chemotherapy regimens on body composition and functional parameters. Moreover, as a retrospective study, certain aspects of patient selection and the quality of the data collected may have been slightly biased. Although we controlled for confounding variables such as age, sex, and treatment line, other unmeasured variables could have influenced the results. Additionally, long-term survival data did not distinguish between causes of death due to the unavailability of detailed mortality records. Finally, bioelectrical impedance analysis (BIA) was conducted using the AKERN Nutrilab device, so the results may vary compared to those obtained with other BIA equipment.

## 5. Conclusions

In conclusion, our results emphasise the value of phase angle and hand grip strength as prognostic indicators in patients with haematologic cancer. Specifically, low standardised PhA values (<−1.65), low PhA values (<3.8° for females and <5.4° for males), and low HGS values (<17 for females and <28 for males) could be predictors of 12-month mortality.

These measures are valuable not only for predicting short-term mortality but also to inform on nutritional and rehabilitation strategies. Incorporating them into clinical practice could improve both the evaluation and management of these patients, leading to better outcomes.

## Figures and Tables

**Table 1:** Demographic parameters, nutritional tools, BIA, functional test and outcome results by survival or non-survival.

Parameters	Total	Survival	Non-Survival	*p* Value
N	121	55 (45.5%)	66 (54.5%)	
Age (years)	64 (19–89)	59.2 (19–83)	70.7 (56–89)	<0.001
Height (cm)	167 (140–187)	168 (140–187)	166 (150–187)	<0.001
Weight (kg)	69.9 (39–120)	71.7 (39–120)	48.2 (48.2–86.2)	<0.001
Weight loss (%)	8.7 (0–32.5)	4.44 (0–32.5)	8.76 (0–29)	0.010
BMI (kg/m^2^)	25.0 (15.4–41.5)	25.4 (15.4–41.5)	24.4 (17.9–32)	0.229
SGA n (%)				<0.001
A	32 (26.4%)	29 (24%)	3 (2.5%)	
B	50 (41.4%)	30 (24.8%)	20 (16.5%)	
C	39 (32.2%)	17 (14%)	22 (18.2%)	
GLIM n (%)				<0.001
No malnutrition	51 (42.1%)	42 (35%)	9 (7.5%)	
Moderate	42 (34.7%)	24 (20%)	18 (15%)	
Severe malnutrition	27 (23.2%)	9 (7.5%)	18 (15%)	
PhA (°)	M 4.95 (3–7.6) F 4.1 (2.1–6.8)	5.34 (3.2–7.6) 4.63 (3–6.8)	4.24 (3–6.2) 3.3 (2.1–5.2)	<0.001
SPhA	−1.1 (−4.6–3.8)	−0.65 (−4.6–3.8)	−1.92 (−4.4–0.7)	<0.001
BCM (kg)	22.1 (9–41.6)	24.4 (10–41.6)	17.7 (9–36.1)	<0.001
SMI (kg/m^2^)	M 9.92 (6.3–13.1) F 7.57 (5.6–10.9)	10.4 (8–13.1) 7.56 (5.6–10.9)	9.06 (6.3–11.8) 7.59 (5.7–9.2)	0.042
HGS (kg)	M 34.4 (15–52) F 20.4 (9.6–30)	36.1 (23–52) 20.8 (9.6–30)	29.6 (15–38) 19.7 (13–28)	0.022
Hospital length of stay (days)	12.4 (2–61)	10.5 (2–45)	15.6 (2–61)	0.024
Line of treatment n				
First	83 (68.6%)	64 (52.9%)	19 (15.7%)	<0.001
Second	38 (31.4%)	12 (9.9%)	26 (21.5%)	

*p* for comparison by gender and line of treatment. Weight loss: percentage of body weight lost in the last 6 months. BMI: body mass index; Subjective Global Assessment (SGA); PhA: phase angle differentiated by sex; SPhA: standardized phase angle; BCM: body cell mass; SMI: Skeletal Muscle Index differentiated by sex; HGS: hand grip strength differentiated by sex; Hospital length of stay: number of days each patient remained hospitalized.

**Table 2:** Rho’s Spearman correlations between hand grip test and phase angle parameters measured by bioelectrical impedance analysis (BIA) at baseline.

	BMI	WL	Albumin	PhA	SPhA	HGS
BMI	-					
Weight loss	0.368 ***	-				
Albumin	−0.002	0.124	-			
PhA	0.202 *	−0.291 **	0.390 ***	-		
SPhA	0.097	−0.189 *	0.273 **	0.810 ***	-	
HGS	0.216	−0.198	0.371 **	0.378 **	0.059	-

Note. * *p* < 0.05, ** *p* < 0.01, *** *p* < 0.001. BMI: body mass index; Weight loss: percentage of body weight lost in the last 6 months; PhA: phase angle; SPhA: standardized phase angle; HGS: hand grip strength.

**Table 3:** Differences of PhA and SPhA values in survival and non-survival patients.

12-Month Mortality			
	Survival Patients	Non-Survival Patients	*p*
PhA	5.0 ± 1.2	3.7 ± 1.1	<0.001
SPhA	−0.6 ± 1.6	−1.9 ± 1.3	<0.001

PhA: phase angle; SPhA: standardized phase angle.

**Table 4:** Diagnostic accuracy of PhA and SPhA to predict 12-month mortality.

Parameters	AUC	Cut-Off Point	Sensitivity	Specificity	PPV	NPV
PhA (°)	0.798	3.8	85.5%	62.2%	79.3%	71.8%
Male	0.762	5.4	55.6%	89.5%	90.9%	51.5%
Female	0.845	3.8	82.5%	76.9%	84.6%	74.1%
SPhA	0.737	−1.5	70.7%	68.9%	79.1%	58.5%

PhA: phase angle; SPhA: standardized phase angle.

**Table 5:** Diagnostic accuracy of HGS to predict 12-months mortality.

Parameters	AUC	Cut-Off Point	Sensitivity	Specificity	PPV	NPV
HGS (Female)	0.568	17 kg	95.24%	28.57%	66.67%	80%
HGS (Male)	0.698	28 kg	88.89%	40%	80%	57.14%

HGS: hand grip strength.

**Table 6:** Binomial logistic regression model for 12-month mortality prediction in HC.

Predictor	Estimate	SE	Z	*p*	Odds Ratio
Constant	2.0671	3.7765	0.547	0.584	7.902
Sex:					
F–M	−0.4491	1.0067	−0.446	0.656	0.638
Age	0.0373	0.0333	1.119	0.263	1.038
Line of treatment:					
2–1	2.6564	0.7509	3.538	<0.001	14.245
PhA	−0.8745	0.3835	−2.280	0.023	0.417
HGS	−0.0893	0.0736	−1.213	0.225	0.915

HGS: hand grip strength; PhA: phase angle. The estimates represent the log odds of “Mortality = 1” vs. “Mortality = 0”.

## Data Availability

The data presented in this study are available on request from the corresponding author due to privacy restrictions at the Quirónsalud Hospital.

## Supplementary Materials

The following supporting information can be downloaded at https://www.mdpi.com/article/10.3390/cancers17050886/s1, Figure S1: flow chart diagram; Table S1: Distribution of Haematological Cancers Subtypes in the Study Population. This table categorizes the haematological cancers subtypes encountered in the patient cohort and outlines the progression of treatment phases among the patients involved in the study; Table S2: Demographic parameters, nutritional tools, BIA, Functional test and Outcomes results by gender and by the line of treatment.

## References

[1] Cazzola M., Sehn L.H. (2022). Developing a Classification of Neoplasms in the Era of Precision Medicine. Blood.

[2] Szeja N., Grosicki S. (2022). Nutritional Status of Patients with Lymphoproliferative Neoplasms before and after the First-Line Treatment. Clin. Nutr..

[3] Cederholm T., Jensen G.L., Correia M.I.T.D., Gonzalez M.C., Fukushima R., Higashiguchi T., Baptista G., Barazzoni R., Blaauw R., Coats A. (2019). GLIM criteria for the diagnosis of malnutrition—A consensus report from the global clinical nutrition community. Clin. Nutr..

[4] Muscaritoli M., Arends J., Bachmann P., Baracos V., Barthelemy N., Bertz H., Bozzetti F., Hütterer E., Isenring E., Kaasa S. (2021). ESPEN practical guideline: Clinical Nutrition in cancer. Clin. Nutr..

[5] Almeida J.M.G., García C.G., Aguilar I.M.V., Castañeda V.B., Guerrero D.B. (2021). Morphofunctional assessment of patient’s nutritional status: A global approach. Nutr. Hosp..

[6] Soeters P.B., Reijven P.L., Schols J.M., Halfens R.J., Meijers J.M., van Gemert W.G. (2008). A rational approach to nutritional assessment. Clin. Nutr..

[7] García-Almeida J.M., García-García C., Ballesteros-Pomar M.D., Olveira G., Lopez-Gomez J.J., Bellido V., Bretón Lesmes I., Burgos R., Sanz-Paris A., Matia-Martin P. (2023). Expert Consensus on Morphofunctional Assessment in Disease-Related Malnutrition. Grade Review and Delphi Study. Nutrients.

[8] Fernández-Jiménez R., Dalla-Rovere L., García-Olivares M., Abuín-Fernández J., Sánchez-Torralvo F.J., Doulatram-Gamgaram V.K., Hernández-Sanchez A.M., García-Almeida J.M. (2022). Phase Angle and Handgrip Strength as a Predictor of Disease-Related Malnutrition in Admitted Patients: 12-Month Mortality. Nutrients.

[9] Garlini L.M., Alves F.D., Ceretta L.B., Perry I.S., Souza G.C., Clausell N.O. (2019). Phase angle and mortality: A systematic review. Eur. J. Clin. Nutr..

[10] Norman K., Stobäus N., Pirlich M., Bosy-Westphal A. (2012). Bioelectrical phase angle and impedance vector analysis–clinical relevance and applicability of impedance parameters. Clin. Nutr..

[11] Cardinal T.R., Wazlawik E., Bastos J.L., Nakazora L.M., Scheunemann L. (2010). Standardized phase angle indicates nutritional status in hospitalized preoperative patients. Nutr. Res..

[12] Bellido D., García-García C., Talluri A., Lukaski H.C., García-Almeida J.M. (2023). Future lines of research on phase angle: Strengths and limitations. Rev. Endocr. Metab. Disord..

[13] de Almeida C., Penna P.M., Pereira S.S., Rosa C.D.O.B., Franceschini S.D.C.C. (2021). Relationship between Phase Angle and Objective and Subjective Indicators of Nutritional Status in Cancer Patients: A Systematic Review. Nutr. Cancer.

[14] Victoria-Montesinos D., García-Muñoz A.M., Navarro-Marroco J., Lucas-Abellán C., Mercader-Ros M.T., Serrano-Martínez A., Abellán-Aynés O., Barcina-Pérez P., Hernández-Sánchez P. (2023). Phase Angle, Handgrip Strength, and Other Indicators of Nutritional Status in Cancer Patients Undergoing Different Nutritional Strategies: A Systematic Review and Meta-Analysis. Nutrients.

[15] Liu M.A., DuMontier C., Murillo A., Hshieh T.T., Bean J.F., Soiffer R.J., Stone R.M., Abel G.A., Driver J.A. (2019). Gait speed, grip strength, and clinical outcomes in older patients with hematologic malignancies. Blood.

[16] Sealy M.J., Dechaphunkul T., van der Schans C.P., Krijnen W.P., Roodenburg J.L.N., Walker J., Jager-Wittenaar H., Baracos V.E. (2020). Low muscle mass is associated with early termination of chemotherapy related to toxicity in patients with head and neck cancer. Clin. Nutr..

[17] Amano K., Bruera E., Hui D. (2023). Diagnostic and prognostic utility of phase angle in patients with cancer. Rev. Endocr. Metab. Disord..

[18] Detsky A.S., McLaughlin J.R., Baker J.P., Johnston N., Whittaker S., Mendelson R.A., Jeejeebhoy K.N. (1987). What is subjective global assessment of nutritional status?. JPEN J. Parenter. Enter. Nutr..

[19] Piccoli A., Nigrelli S., Caberlotto A., Bottazzo S., Rossi B., Pillon L., Maggiore Q. (1995). Bivariate normal values of the bioelectrical impedance vector in adult and elderly populations. Am. J. Clin. Nutr..

[20] Genton L., Herrmann F.R., Spörri A., Graf C.E. (2018). Association of mortality and phase angle measured by different bioelectrical impedance analysis (BIA) devices. Clin. Nutr..

[21] Paiva S.I., Borges L.R., Halpern-Silveira D., Assunção M.C.F., Barros A.J.D., Gonzalez M.C. (2011). Standardized phase angle from bioelectrical impedance analysis as prognostic factor for survival in patients with cancer. Support. Care Cancer.

[22] Arab A., Karimi E., Vingrys K., Shirani F. (2021). Is phase angle a valuable prognostic tool in cancer patients’ survival? A systematic review and meta-analysis of available literature. Clin. Nutr..

