# Supplementary material for: Role of Bioimpedance Phase Angle and Hand Grip Strength in Predicting 12-Month Mortality in Patients Admitted with Haematologic Cancer
Source: Cancers (Basel). 2025 Mar 5;17(5):886. doi: 10.3390/cancers17050886 (PMC11898618; doi:10.3390/cancers17050886)
Supplement: Supplementary file 1 [file cancers-17-00886-s001.zip › cancers-3473616-supplementary.pdf]

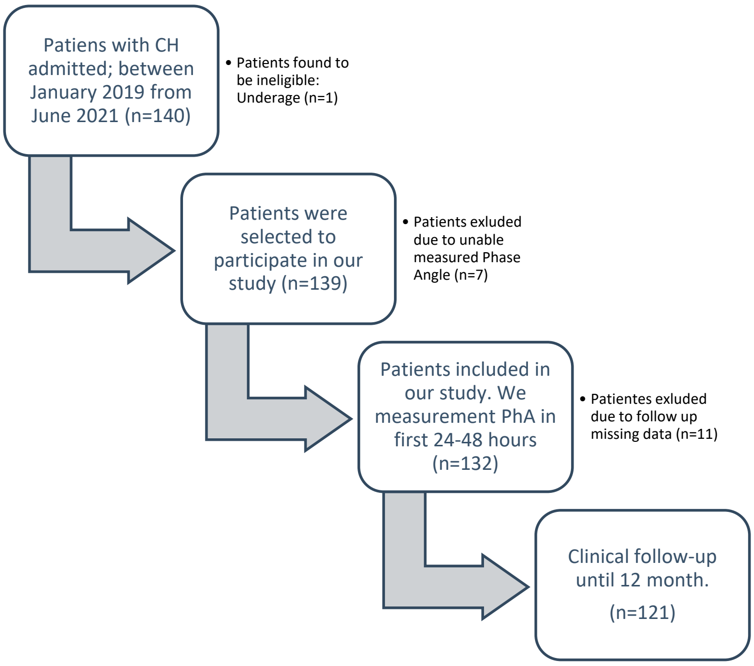

Supplementary Figure S1. Flow chart diagram.

Supplementary Table S1. Distribution of Haematological Cancer Subtypes in the Study Population. This table categorizes the haematological cancer subtypes encountered in the patient cohort and outlines the progression of treatment phases among the patients involved in the study.

Haematological Cancer Subtype	***Total (%)***
Follicular lymphoma (FL)	10 (8.5%)
Acute Myeloid Leukemia (LMA)	26 (22%)
Burkitt's Lymphoma (LNH-Burkitt)	2 (1.7%)
Chronic Lymphocytic Leukemia (LLC)	2 (1.7%)
Non-Hodgkin's Lymphoma (LNH)	4 (3.4%)
T/NK-cell Lymphoma (LNH-T/NK)	8 (6.7%)
Myeloma	20 (16.8%)
Acute Lymphoblastic Leukemia (LLA)	4 (3.4%)
Hodgkin's Lymphoma (LH)	1 (0.8%)
Diffuse Large B-Cell Lymphoma (LDCBG)	30 (25.4%)
Mantle Cell Lymphoma (MCL)	4 (3.4%)
MALT Lymphoma	2 (1.7%)
Amyloidosis	4 (3.4%)
Nodal Marginal Zone Lymphoma (NMPC)	1 (0.8%)
Treatment Phase	***Total (%)***
Initial Treatment	27 (22.5%)
Remission	55 (45.8%)
Relapse/Refractory	38 (31.7%)

Supplementary Table S2. Demographic parameters, nutritional tools, BIA, Functional test and Outcomes results by gender and by the line of treatment.

Parameters	*Total*	*Male patients*	*Female patients*	*P value*
N	121	55 (45.5%)	66 (54.5%)	
Age (years)	64 (19-89)	64 (27-83)	63 (19-89)	0.306
Height (cm)	167 (140-187)	173 (158-187)	162 (140-175)	<0.001
Weight (kg)	69.9 (39-120)	76.6 (56-102)	64.3 (39-120)	<0.001
Loss weight (%)	8.7 (0-32.5)	6.3 (0-20)	10.9 (0-32.5)	0.324
BMI (kg/m^2)	25.0 (15.4-41.5)	25.8 (17.9-36.9)	24.4 (15.4-41.5)	0.008
SGA n (%)				0.002
A	32 (26.4%)	20 (36.4%)	12 (18.2%)	
B	50 (41.4%)	26 (47.3%)	24 (36.4 %)	
C	39 (32.2%)	9 (16.4%)	30 (45.5%)	
GLIM n (%)				0.333
No malnutrition	51 (42.1%)	25 (45.5%)	26 (39.4%)	
Moderate	42 (34.7%)	21 (38.2%)	21 (31.8%)	
Severe malnutrition	27 (23.2%)	9 (16.3%)	18 (27.3%)	
PhA (°)	4.5 (2.1-7.6)	5.0 (3-7.6)	4.1 (2.1-6.8)	<0.001
SPhA	-1.1 (-4.6-3.8)	-1.2 (-4.6-1.9)	-1 (-4.4-3.8)	0.556
BCM (kg)	22.1 (9-41.6)	27.2 (15.6-41.6)	18.0 (9-36.2)	<0.001
HGS (kg)	27.4 (9.6-52)	34.2 (15-52)	20.4 (9.6-30)	<0.001
Long stay (days)	12.4 (2-61)	10.2 (2-45)	14.3 (2-61)	0.094
Death n (%)	45 (37.5%)	19 (15.8%)	26 (21.7%)	0.583

Parameters	*Total*	*First Line*	*Second Line*	*P value*
N	121	83 (68.6%)	38 (31.4%)	
Sex n (%)	M 55 (45.5%)<br>F 66 (54.4%)	37 (44.6%)<br>46 (55.4%)	18 (47.4%)<br>20 (52.6%)	0.775
Age (years)	64 (19-89)	61.7 (19-89)	67.2 (27-86)	0.228
Weight (kg)	69.9 (39-120)	70.7 (39-120)	68.2 (48.2-102)	0.251
Loss weight (%)	8.7 (0-32.5)	7.97 (0-32.5)	10.2 (0-29)	0.003
BMI (kg/m^2)	25.0 (15.4-41.5)	25.1 (15.4-41.5)	24.8 (17.9-36.9)	0.441
SGA n (%)				0.037
A	32 (26.4%)	26 (31.3%)	6 (15.8%)	
B	50 (41.4%)	36 (43.4%)	14 (36.8 %)	
C	39 (32.2%)	21 (25.3%)	18 (47.4%)	
GLIM n (%)				0.333
No malnutrition	51 (42.1%)	42 (45.5%)	9 (40%)	
Moderate	42 (34.7%)	29 (38.2%)	13 (23.3%)	
Severe malnutrition	27 (23.2%)	11 (16.4%)	16 (27.7%)	
PhA (°)	4.5 (2.1-7.6)	4.7 (2.1-7.8)	4.1 (2.3-6.6)	0.015
SPhA	-1.1 (-4.6-3.8)	-0.87 (-4.6-3.8)	-1.6 (-4.4-1.1)	0.028
BCM (kg)	22.1 (9-41.6)	23.1 (9.4-40.6)	19.9 (9-41.6)	0.041
HGS (kg)	27.4 (9.6-52)	27.9 (9.6-52)	26.9 (13-51)	0.867
Long stay (days)	12.4 (2-61)	11.4 (2-61)	14.8 (2-57)	0.257
Death n (%)	45 (37.5%)	19 (22.9%)	26 (68.4%)	<0.001

p for comparison by gender and line of treatment. BMI body mass index; Subjective Global Assessment (SGA); PhA: phase angle; SPhA: standardized phase angle; BCM: body cell mass, HGS: handgrip strength.
